# Combining Network Pharmacology and Experimental Verification to Investigate the Protective Effect of Melatonin on Fluoride-Induced Brain Injury

**DOI:** 10.3390/toxics14020128

**Published:** 2026-01-29

**Authors:** Runjiang Ma, Chun Wang, Wenqi Qin, Yajie Li, Meng Zhang, Yongkang Liang, Lu Wang, Suya Wang, Gaoxuan Xie, Qiang Niu

**Affiliations:** 1 School of Public Health, Shihezi University, Shihezi 832000, China; 18194615414@163.com (R.M.); 18399728867@163.com (C.W.); yh13047155642@163.com (W.Q.); 19967109963@163.com (Y.L.); 15609921586@163.com (M.Z.); xxrtyyds66@163.com (Y.L.); qq3480534563@163.com (L.W.); 17799931107@163.com (S.W.); 17613680889@163.com (G.X.); 2 Key Laboratory for Prevention and Control of Emerging Infectious Diseases and Public Health Security, Shihezi 832000, China; 3 Key Laboratory of Xinjiang Endemic and Ethnic Diseases (Ministry of Education), School of Medicine, Shihezi University, Shihezi 832000, China

**Keywords:** melatonin, sodium fluoride, neurotoxicity, SIRT3/HIF-1α, energy metabolism, network pharmacology

## Abstract

Excessive fluoride exposure induces developmental neurotoxicity, but effective preventive strategies are currently scarce. Melatonin (Mel), a lipophilic hormone secreted by the pineal gland, exerts antioxidant, anti-inflammatory, and neuroprotective properties. This study aimed to explore Mel’s protective effect and mechanism against fluoride-induced developmental brain injury. We employed a network pharmacology approach to screen the common targets of Mel and fluoride-induced brain injury and performed enrichment analysis. A total of 189 common targets were identified, and these targets were mainly enriched in the HIF-1 signaling pathway and oxidative stress-related pathways. In vivo, Sprague Dawley rats were subjected to perinatal sodium fluoride (NaF) exposure with/without Mel; in vitro, HT22 cells were subjected to NaF and/or Mel. The results showed that Mel improved cognitive impairments and alleviated structural damage to hippocampal neurons and mitochondria. Furthermore, Mel upregulated SIRT3 and downregulated HIF-1α, thereby restoring mitochondrial oxidative phosphorylation and ATP content. This study demonstrates that Mel alleviates fluoride-induced developmental neurotoxicity by improving mitochondrial function through regulating the SIRT3/HIF-1α signaling pathway. This not only offers a novel perspective for elucidating the underlying molecular mechanisms of fluoride-induced developmental neurotoxicity but also provides a theoretical foundation for Mel as a potential protective candidate against fluoride exposure.

## 1. Introduction

Fluorine is naturally present in the environment, possessing high electronegativity and reactive chemical properties, capable of combining with various elements to form compounds such as hydrofluoric acid and sodium fluoride [1]. The fluoride concentration in groundwater far exceeds the standard of 1.5 mg/L in many countries and regions, including China, Africa, South Asia, and the Middle East [2]. Fluoride primarily enters the human body through the digestive and respiratory tracts. Adequate fluoride intake prevents dental caries and enhances bone strength effectively, while excessive exposure causes multisystem damage to the brain, kidneys, and liver [3]. Research indicates that the embryonic developmental period is a highly sensitive stage for fluoride exposure, potentially causing lifelong bodily harm [4]; furthermore, puberty is an important period characterized by elevated susceptibility of the organism to fluoride and other environmental toxins [5,6]. Epidemiological studies and animal experiments demonstrate that early-life fluoride exposure can induce postnatal learning and cognitive impairments [1,7]. Fluoride-induced neurotoxicity is mediated through multiple interconnected pathways, involving abnormal cell morphology and cytoskeletal protein expression, inflammatory responses, oxidative stress, neurotransmitter and related enzyme dysfunction, autophagy impairment, ferroptosis, and mitochondrial toxicity [8,9]. In recent years, research into the mechanisms underlying fluoride-induced neurotoxicity has garnered considerable attention. Yet, the complete pathophysiological process remains to be fully elucidated, and more critically, effective strategies to mitigate the associated health risks are lacking.

A growing body of evidence has pinpointed mitochondrial dysfunction as a central event in fluoride-induced neurotoxicity, given that fluoride can disrupt cellular energy metabolism processes to trigger neuronal damage. On the one hand, fluoride impairs mitochondrial ultrastructure and alters its permeability, inhibits the activity of tricarboxylic acid cycle enzymes, impairs mitochondrial respiratory chain function, and consequently reduces intracellular adenosine triphosphate (ATP) levels, ultimately inducing cellular apoptosis [10]. On the other hand, fluoride exposure downregulates the expression of mitochondrial deacetylases, resulting in hyperacetylation of downstream superoxide dismutase and marked accumulation of intracellular reactive oxygen species (ROS) [11]; excess ROS induces oxidative damage to mitochondrial proteins and lipids, further impairing mitochondrial electron transport chain function, exacerbating mitochondrial energy metabolism dysfunction, and ultimately triggering neuronal death [12]. Although antioxidants [13] have been proven to alleviate fluoride-induced neurotoxicity to some extent, their therapeutic efficacy is limited by the multi-pathway nature of fluoride toxicity. This highlights the urgent need to identify safe and effective agents that can target multiple pathological links to counteract fluoride-induced neurotoxicity.

Melatonin (Mel), a lipophilic small-molecule hormone predominantly secreted by the pineal gland and characterized by nocturnal rhythmic secretion, exerts diverse pharmacological effects in organisms. Mel exhibits extensive pharmacological properties encompassing antioxidant, anti-inflammatory, immunomodulatory, and anticancer activities [14], and exerts neuroprotective effects via multiple pathways, namely regulating cytokine secretion, mitigating oxidative stress, and preserving mitochondrial function [15]. In recent years, Mel has been widely applied in the treatment of neurodegenerative diseases. Studies have demonstrated that Mel exerts protective effects not only against neurodegenerative diseases, including Alzheimer’s disease [16] and Parkinson’s disease [17], but also against brain damage induced by environmental toxicants such as nickel [18] and polybrominated diphenyl ethers [19]. However, the precise molecular targets and core pathways mediating the protective effects of melatonin against fluoride-induced developmental neurotoxicity remain unclear, a knowledge gap that can be addressed by the systematic analytical framework of network pharmacology.

Network pharmacology is an emerging interdisciplinary methodology that integrates diverse technical tools to investigate drug–disease correlations and employs network visualization techniques to identify key molecular targets and core active components of drugs. To address the aforementioned knowledge gap, the present study aimed to explore Mel’s protective effect and its underlying molecular mechanism against fluoride-induced developmental brain injury. Based on this methodology, we established a perinatal sodium fluoride (NaF) exposure model with/without Mel intervention, employed network pharmacology approaches to predict key molecular targets of Mel for treating fluoride-induced nerve injury, and integrated experimental assays to elucidate the therapeutic effects and underlying mechanisms of Mel against fluoride-induced nerve injury.

Our results showed that a total of 189 common targets were identified through network pharmacology analysis, and these targets were mainly enriched in the HIF-1 signaling pathway and oxidative stress-related pathways. In vivo experiments on Sprague Dawley rats and in vitro assays on HT22 cells confirmed that Mel improved cognitive impairments and alleviated structural damage to hippocampal neurons and mitochondria. Furthermore, Mel was found to upregulate SIRT3 and downregulate HIF-1α, thereby restoring mitochondrial oxidative phosphorylation and increasing ATP content. Collectively, this study confirms that Mel exerts a protective effect against fluoride-induced developmental neurotoxicity and further elucidates the underlying key molecular pathways mediating its therapeutic efficacy. Furthermore, the present study’s findings provide a theoretical basis for Mel as a potential protective agent against environmental fluoride exposure.

## 2. Materials and Methods

### 2.1. Antibodies and Reagents

NaF (7681-49-4) and Mel (M5250) were obtained from Sigma (Ronkonkoma, NY, USA). Hypoxia-inducible factor 1 alpha (HIF-1α) (TA1009S) antibody was purchased from Abmart (Shanghai, China); NAD-dependent deacetylase sirtuin-3 (SIRT3) (10099-1-AP) and NADH dehydrogenase ubiquinone Fe-S protein 1 (NDUFS1) (12444-1-AP) antibodies were supplied by Proteintech (Rosemont, IL, USA). The Cell Counting Kit-8 (CCK-8, BS350B) was purchased from Biosharp (Beijing, China). The ATP assay kit (S0027) was purchased from Beyotime (Shanghai, China). The mitochondrial membrane potential assay kit (JC-1, M8650), RIPA lysis buffer (R0010), ROS assay kit (CA1410), and dimethyl sulfoxide (D8371) were provided by Solarbio (Beijing, China).

### 2.2. Animals and Treatments

Adult Sprague Dawley (SD) rats were purchased from SPF Biotechnology Co., Ltd. (Beijing, China), license number: SCXK (Jing) 2024-0001. Rats were housed in the SPF animal facility of Shihezi University under standard conditions (20–25 °C, 50–60% humidity, 12 h light/dark cycle). The experimental protocol was approved by the Animal Experimental Ethics Committee of the First Affiliated Hospital, Shihezi University School of Medicine (Approval No.: A2023-062-01), and conducted in strict compliance with the NIH Guide for the Care and Use of Laboratory Animals.

After one week of acclimatization, thirty-six SD rats were randomly assigned to 6 groups (female–male = 2:1) for cohabitation. NaF was given by gavage, and Mel by intraperitoneal injection. The experimental groups were as follows: control group (administered double-distilled water), NaF group (40 mg/kg/day), Mel group (10 mg/kg/day), and combined NaF and Mel group (40 mg/kg/day NaF and 10 mg/kg/day Mel). Pregnant females were singly housed and continued their respective treatments. Offspring received intraperitoneal Mel (10 mg/kg/day) [19] on postnatal day 8; from postnatal day 10 onward, they were treated with the same route and dose as their parents up to 2 months of age.

### 2.3. Cell Culture and Treatment

HT22 cells were cultured in Dulbecco’s modified Eagle’s medium (DMEM) supplemented with 10% fetal bovine serum (FBS, Biological Industries, Kibbutz Beit Haemek, Israel) and 1% penicillin–streptomycin (Solarbio), and maintained in a humidified incubator at 37 °C with 5% CO_2_. NaF was dissolved in double-distilled water to prepare a 4 g/L stock solution, which was diluted to the working concentration prior to use. When HT22 cells reached 70–80% confluence, they were treated with 60 mg/L NaF and maintained in incubation for an additional 24 h. The exposure dose was determined based on previous research [20]. Mel powder was dissolved in DMSO and diluted to 20 μmol/L in DMEM prior to treatment. At 70–80% cell confluence, cells were pretreated with 20 μmol/L Mel for 2 h; the concentration was determined by CCK-8 assays (Figure S1A,B) and prior literature [21].

### 2.4. Morris Water Maze (MWM) Test

The Morris water maze (MWM) was performed to assess spatial learning and memory in rats: A four-day place navigation trial was conducted, during which each rat was placed into the water from four different quadrants of the pool each day, and the time required to find the submerged, hidden platform was recorded. Rats that failed to find the platform within 60 s were gently guided to it and allowed to remain there for 15 s. On the fifth day, a spatial probe trial was performed with the platform removed from the pool. Rats were allowed to swim for 60 s, during which the number of platform crossings, swimming distance in the target quadrant, and time spent in the target quadrant were recorded.

### 2.5. Observation of Mitochondrial Ultrastructure in Hippocampal Tissue by Transmission Electron Microscopy (TEM)

After euthanasia, the brain was removed, and the hippocampus was dissected and cut into approximately 1 mm^3^ tissue blocks. These tissue blocks were fixed in an electron microscopy fixative at 4 °C for 24 h. Subsequently, the samples were sequentially dehydrated through a graded ethanol series (50%, 70%, 90%, and 100%), infiltrated with acetone, and then embedded in epoxy resin. After the resin had fully polymerized, the embedded blocks were sectioned into 50 nm thick slices using an ultramicrotome. The sections were stained with 4% uranyl acetate for 20–25 min and 0.5% lead citrate for 8–10 min and then observed under a Hitachi HT7800 transmission electron microscope (Hitachi High-Tech, Tokyo, Japan).

### 2.6. Western Blot

Total proteins were extracted from rat hippocampal tissue and HT22 cells using RIPA lysis buffer. Protein concentration was determined using the biuret reaction in the BCA modification. The molecular weight marker (G2058) was supplied by Servicebio (Wuhan, China). Protein samples were separated by 10% SDS-PAGE gel electrophoresis and then transferred onto PVDF membranes. The membranes were blocked with 5% non-fat milk at room temperature for 1.5 h, washed three times, and then incubated with primary antibodies overnight at 4 °C. Primary antibodies were used at the following dilutions: SIRT3 (1:1000), HIF-1α (1:1000), NDUFS1 (1:5000), β-actin (1:50,000), and GAPDH (1:50,000). Subsequently, the membranes were incubated with a secondary antibody at room temperature for 1.5 h. The membranes were washed three times with Tris-buffered saline containing Tween 20, after which protein bands were visualized by enhanced chemiluminescence (ECL) reagent using a Tanon-5200 chemiluminescence imaging analysis system. Quantification was performed using ImageJ 1.54g software, and each experiment was repeated three times independently.

### 2.7. Mitochondrial Membrane Potential (MMP) and ROS Levels Assay

The JC-1 probe was used to detect MMP levels. HT22 cells were treated with NaF, washed with PBS, and then cells in the positive control group received 10 μM CCCP (carbonyl cyanide 3-chlorophenylhydrazone)—a positive control for inducing MMP dissipation. The cells were incubated in the culture incubator for 20 min; then, a JC-1 staining working solution was added to each group, followed by further incubation for 20 min. After incubation, cells were gently rinsed thrice with pre-cooled 1× staining buffer, refilled with fresh DMEM, and immediately visualized under a laser scanning confocal microscope (Nikon AXR, Tokyo, Japan) in dark conditions for fluorescence intensity assessment. Quantification was conducted using ImageJ software, and all experiments were repeated three times independently. Background subtraction of fluorescence images was performed using ImageJ software, followed by normalization against the mean fluorescence intensity of the control group; the final results are expressed as relative fluorescence intensity.

Cells were seeded in 6-well plates and cultured in a cell incubator. Upon reaching the desired confluence, the medium was aspirated, and cells were rinsed once with PBS prior to experimental treatment. After 24 h of treatment, cells were washed with PBS, digested with EDTA-free 0.25% trypsin, and harvested by centrifugation. DCFH-DA (1:8000 dilution in DMEM) was added to the collected cells; after 20 min incubation, ROS levels were measured by flow cytometry. All experiments were independently repeated in triplicate. In this experiment, the background signal baseline was determined with a negative control tube, and FlowJo 10.10.0 software was applied to perform background subtraction on the raw signals from the flow cytometer, ultimately yielding the mean fluorescence intensity for each group.

### 2.8. ATP Levels’ Assay

ATP was extracted from tissue samples by adding 100–200 µL of lysis buffer per 20 mg of tissue, while cell samples were lysed with 200 µL of lysis buffer per well. ATP levels were subsequently measured in a 96-well plate using a bioluminescence assay. A volume of 100 µL of ATP assay working solution was added to each assay well and incubated at room temperature for 3–5 min. Then, 30 µL of each sample was added to the corresponding well, mixed rapidly, and the relative light units (RLUs) were recorded using the chemiluminescence mode of a multi-mode microplate reader with an interval of at least 2 s between readings. All measurements were performed in three independent experiments.

### 2.9. Energy Metabolism Analysis

The oxygen consumption rate (OCR) was determined using a Seahorse XFe96 Analyzer (Agilent Technologies, Santa Clara, CA, USA). HT22 cells were seeded in XFe96 cell culture microplates at a density of 10,000 cells per well and cultured overnight. OCR was measured under basal conditions; the sequential addition of 1.5 μM oligomycin, 2 μM FCCP, and 0.5 μM rotenone/antimycin A allowed for the determination of mitochondrial basal respiration, ATP production, maximal respiration, and spare respiratory capacity. The value before adding oligomycin represents the basal OCR of the cell, which includes mitochondrial ATP-coupled oxidative phosphorylation respiration and proton leak. Oligomycin, a specific inhibitor of mitochondrial ATP synthase (Complex V), causes a decrease in OCR; this decrease corresponds to the oxygen consumption rate associated with ATP-coupled oxidative phosphorylation, indirectly reflecting the cellular ATP production under this condition. FCCP is an uncoupler that disrupts the proton gradient and mitochondrial membrane potential, thereby uncoupling oxidative phosphorylation from ATP synthesis, which allows electron transport to proceed unrestricted, driving the basal cellular OCR to its maximum, known as the maximal respiratory capacity (a key indicator for assessing mitochondrial functional reserve). Rotenone is a specific inhibitor of mitochondrial Complex I, and antimycin A is a specific inhibitor of mitochondrial Complex III. Co-administration of the two inhibitors potently abrogates mitochondrial respiratory chain electron transport, allowing the measurement of oxygen consumption derived from non-mitochondrial activities in the system. After adding each compound, three measurement cycles were run (4 min mixing, 3 min measurement per cycle). Data were analyzed using Wave Desktop 2.6 software (Agilent Technologies) and normalized to total cell number or total protein content.

### 2.10. Nissl Staining

The paraffin-embedded tissue blocks were sectioned at 4 μm thickness, dewaxed with xylene and absolute ethanol, rehydrated through a graded ethanol series, and then rinsed with distilled water for 2 min. Sections were stained with 1% toluidine blue solution at 60 °C for 40 min, rinsed three times with distilled water, dehydrated in absolute ethanol, cleared in xylene, air-dried, mounted, and observed under a light microscope.

### 2.11. Network Pharmacology

The SMILES information and 3D structure of Mel were retrieved from the PubChem database (https://pubchem.ncbi.nlm.nih.gov) (accessed on 19 October 2025). The 3D structure and SMILES were uploaded to the SwissTargetPrediction (http://www.swisstargetprediction.ch) (accessed on 20 October 2025) and PharmMapper (https://www.lilab-ecust.cn/pharmmapper/) (accessed on 20 October 2025) databases, respectively. Potential targets of Mel were further identified by integrating results from the TCMSP (https://www.tcmsp-e.com/load_intro.php) (accessed on 23 October 2025), DrugBank (https://go.drugbank.com) (accessed on 23 October 2025), and CTD (https://ctdbase.org) (accessed on 24 October 2025) databases. Gene targets related to fluoride-induced brain injury were identified and screened from GeneCards (https://www.genecards.org) (accessed on 25 October 2025), OMIM (https://omim.org) (accessed on 25 October 2025), and TTD (https://ttd.idrblab.cn) (accessed on 25 October 2025) databases. All target genes were standardized using the UniProt database (https://www.uniprot.org) (accessed on 28 October 2025). R 4.3.3 software was used to identify the intersection of fluoride-induced brain injury targets and Mel targets, which were defined as potential therapeutic targets. Protein–protein interaction (PPI) analysis was performed using the STRING database (https://www.string-db.org) (accessed on 5 November 2025) with the species set to “Homo sapiens” and a confidence score threshold ≥ 0.9, to construct a PPI network of targets related to fluoride-induced brain injury and Mel. Key targets were identified from the network using various topological algorithms within the Cytoscape 3.10.1 software. Gene Ontology (GO) and Kyoto Encyclopedia of Genes and Genomes (KEGG) pathway enrichment analyses were conducted using the Metascape database (http://metascape.org) (accessed on 20 November 2025) to elucidate the underlying mechanisms in terms of biological processes, cellular components, molecular functions, and key signaling pathways [22]. The results of the GO and KEGG analyses were visualized using an online bioinformatics platform (http://www.bioinformatics.com.cn) (accessed on 20 November 2025).

### 2.12. Statistical Analyses

Data were analyzed using SPSS 26.0 software, and experimental results are expressed as mean ± standard deviation (SD). The Shapiro–Wilk test and Brown–Forsythe test were used to verify the normality and homogeneity of variances of the data, respectively. One-way analysis of variance (ANOVA) was performed, followed by Tukey’s post hoc test for multiple comparisons. A *p*-value < 0.05 was considered statistically significant.

## 3. Results

### 3.1. Prediction of Potential Targets and Construction of the PPI Network for Mel Alleviating NaF-Induced Brain Damage

**Figure 1:**
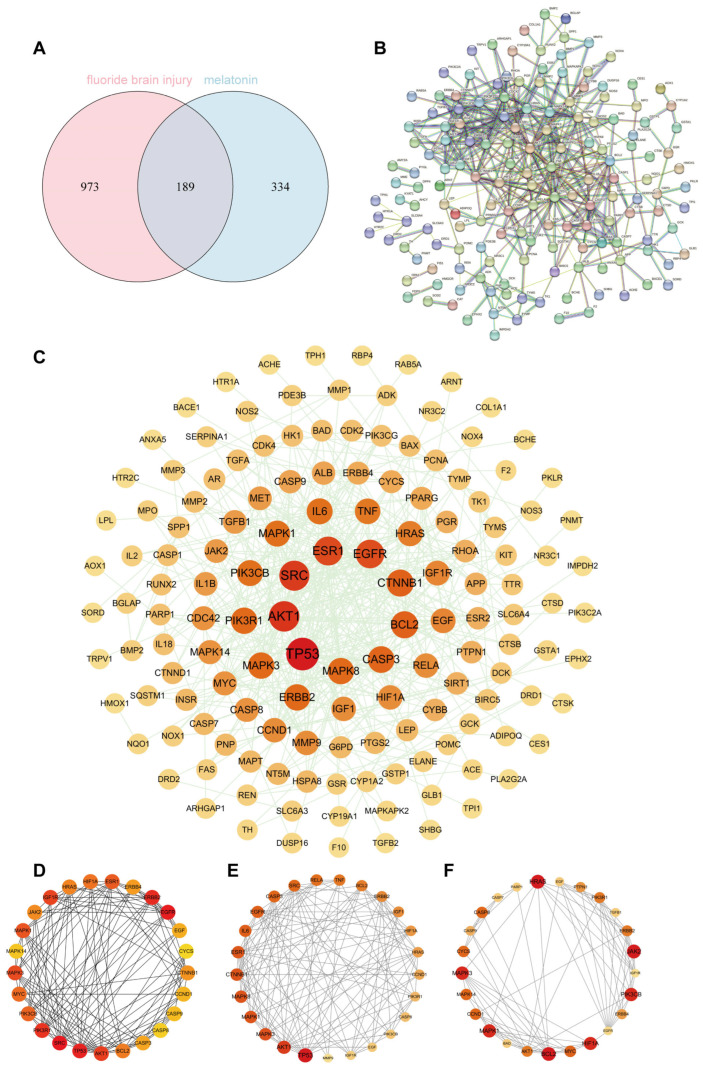
Exploring melatonin-associated proteins for protecting NaF-induced brain injury via network pharmacology: (**A**) Venn diagram of intersection genes of Mel, fluoride, and brain injury. (**B**) PPI network of intersection genes based on the STRING database. (**C**) Network of common targets of Mel and fluoride-induced brain injury. (**D**) Key targets (top 25) screened via the MCC algorithm in Cytoscape. (**E**) Key targets (top 25) screened via CytoNCA in Cytoscape. (**F**) Key targets (top 25) screened via MCODE in Cytoscape. Mel, melatonin; NaF, sodium fluoride; PPI, protein–protein interaction; STRING, Search Tool for the Retrieval of Interacting Genes/Proteins; MCC, Maximal Clique Centrality; Cytoscape, a software for network visualization and analysis; CytoNCA, a Cytoscape app for network centrality analysis; MCODE, Molecular Complex Detection.

To investigate the potential molecular mechanisms by which Mel treats fluoride-induced brain injury, this study employed network pharmacology analysis methods. The potential target genes of Mel were retrieved from the SwissTargetPrediction, PharmMapper, TCMSP, DrugBank, and CTD databases. Following integration and deduplication, a total of 523 valid target genes were identified (Table S1). The target genes associated with fluoride-induced brain injury were retrieved from the GeneCards, OMIM, and TTD databases. Following merging and deduplication, a total of 1162 valid target genes were identified (Table S2). By drawing a Venn diagram, 189 potential overlapping genes were identified from the active targets of Mel and the targets associated with fluoride-induced brain injury (Figure 1A). Next, a PPI network diagram of the intersecting targets was constructed using the STRING database (Figure 1B), with a confidence score threshold set to ≥0.9. Isolated targets were removed, and the processed data were imported into Cytoscape software for visualization and further analysis (Figure 1C). Additionally, this study employed three algorithms from Cytoscape software—Maximum Clique Centrality (MCC), Molecular Complex Detection (MCODE), and CytoNCA—to identify the top 25 core genes (Figure 1D–F).

### 3.2. Analyses of Pathway Enrichment

**Figure 2:**
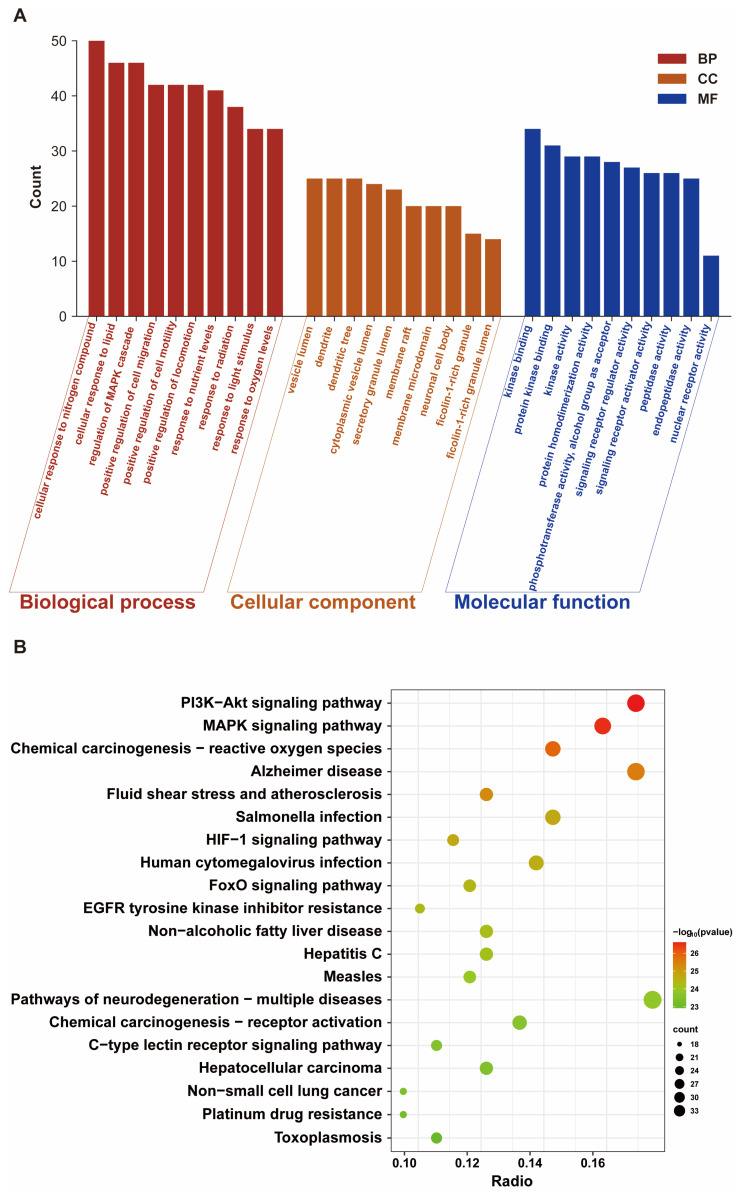
GO and KEGG enrichment analysis of intersection genes: (**A**) Histogram of the top 10 enriched pathways in BP, CC, and MF. (**B**) Bubble plot showing KEGG pathway enrichment of intersecting genes. GO, Gene Ontology; KEGG, Kyoto Encyclopedia of Genes and Genomes; BP, biological process; CC, cellular component; MF, molecular function.

To elucidate the roles of potential therapeutic targets in gene functions and signaling pathways, the candidate therapeutic targets of Mel for fluoride-induced brain injury were submitted to the Metascape platform [22] for GO functional annotation and KEGG pathway enrichment analyses. The GO functional enrichment analysis included 2054 biological processes (BPs), 127 cellular components (CCs), and 197 molecular functions (MF). In BPs, the main enriched terms were mainly involved in cellular responses to nitrogen-containing molecules and lipids, regulation of MAPK cascade, and responses to oxygen levels; in CCs, the main enriched components were primarily localized to vesicle lumen, membrane microdomain, and neuronal cell body; in MFs, the key enriched molecular functions mainly involved protein kinase binding, signal receptor agonist activity, and nuclear receptor activity (Figure 2A). Combined with the aforementioned functional enrichment results, these findings suggest that Mel may exert its protective effects against fluoride-induced brain injury by regulating biological processes such as responses to environmental stimuli, cellular signaling, receptor activation, enzyme activity, and energy metabolism. Additionally, the KEGG analysis identified 208 significantly enriched pathways, mainly including the PI3K-AKT signaling pathway, MAPK signaling pathway, HIF-1 signaling pathway, and chemical carcinogenesis—ROS signaling pathways (Figure 2B). In summary, Mel may protect against fluoride-induced brain injury by regulating neuronal oxidative stress, mitochondrial function, and their related energy metabolism.

### 3.3. Mel Prevents NaF-Induced Cognitive Impairments in Offspring Rats

**Figure 3:**
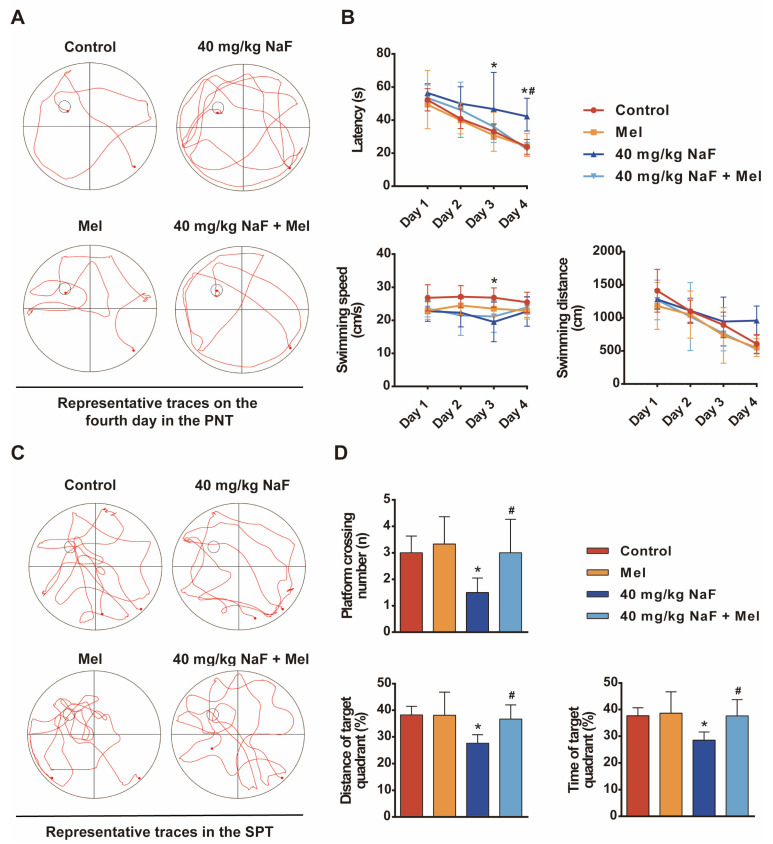
Melatonin prevents NaF-induced cognitive impairment in offspring rats: (**A**) Representative trajectory plot in the PNT. (**B**) The time spent to find the platform, the distance traveled, and the swimming speed in the PNT. (**C**) Representative swimming paths in the SPT. (**D**) The number of platform crossings, the percentage of distance, and the time taken in the goal quadrant in the SPT. Data are shown as mean ± SD (*n* = 6 rats/group). * indicates *p* < 0.05 compared with the control group; ^#^ indicates *p* < 0.05 compared with the 40 mg/kg NaF group. Mel, melatonin; NaF, sodium fluoride; PNT, Place Navigation Test; SPT, Spatial Probe Test.

The MWM test was performed to assess escape latency, time spent in the target quadrant, and other related behavioral indicators, in order to evaluate the protective effect of Mel on NaF-induced cognitive impairment in offspring. Specifically, during the PNT (Place Navigation Test) phase, compared with the NaF group, rats treated with Mel showed a statistically significant reduction in escape latency (Figure 3B, *p* < 0.05), indicating improved spatial learning and memory ability. In the subsequent SPT (Spatial Probe Test) phase, consistent with the PNT results, Mel-treated rats exhibited a significant increase in the number of target platform crossings, as well as a marked elevation in both the percentage of time spent and swimming distance in the target quadrant (Figure 3D, *p* < 0.05). The results showed that Mel significantly alleviated NaF-induced cognitive impairment in offspring rats (Figure 3), which laid a behavioral foundation for further exploring its underlying neuroprotective mechanisms.

### 3.4. Mel Attenuates NaF-Induced Hippocampal Neuronal Damage and Mitochondrial Impairment

**Figure 4:**
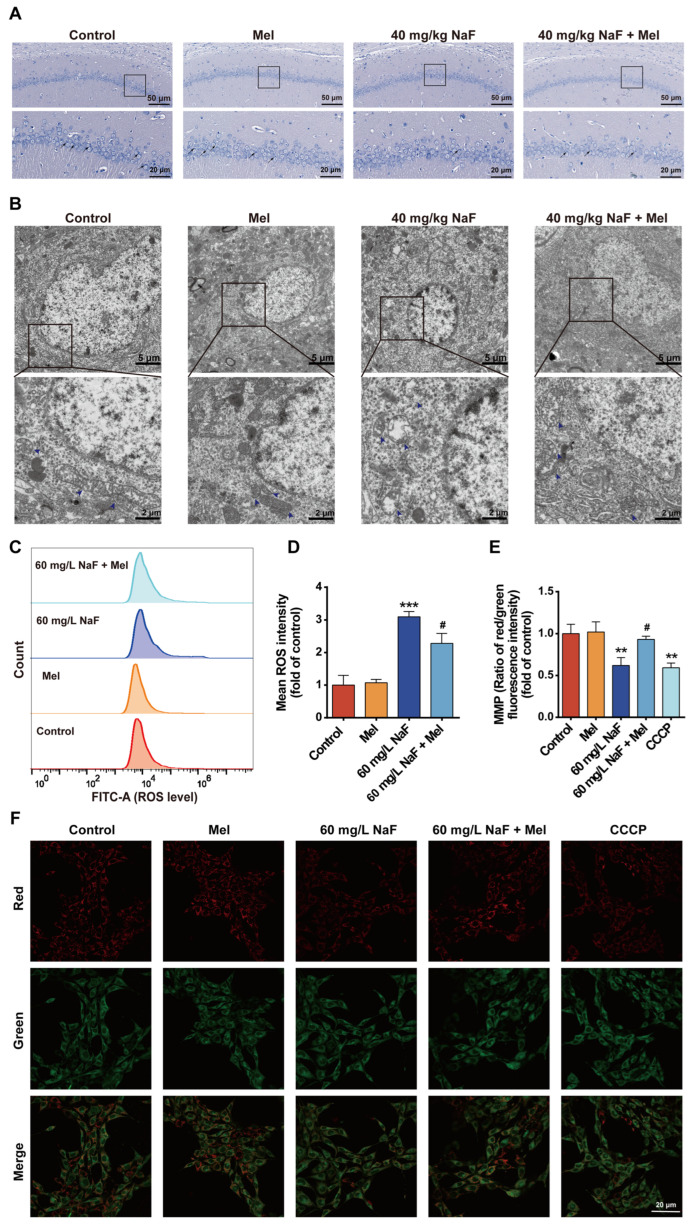
Melatonin attenuates NaF-induced hippocampal neuronal damage and mitochondrial impairment: (**A**) Nissl staining of the CA1 region of the hippocampus in offspring rats. The black arrow indicates the Nissl bodies. (**B**) TEM images of hippocampal neurons in offspring rats. *n* = 3 rats/group. Scale bar, 5 μm (upper row), 2 μm (bottom row); blue arrows indicate mitochondria. (**C**) Flow cytometry plot of ROS levels in HT22 cells exposed to NaF. (**D**) Quantitative analysis of (**C**). (**E**) Quantitative analysis of (**F**). (**F**) Representative images of MMP in HT22 cells. Red signals indicate JC-1 aggregates, green signals indicate JC-1 monomers, and CCCP was used as a positive control. Values represent the mean ± SD (*n* = 3 replicates); ** indicates *p* < 0.01, *** *p* < 0.001 compared with the control group; ^#^ indicates *p* < 0.05, compared with the 60 mg/L NaF group. Mel, melatonin; NaF, sodium fluoride; TEM, transmission electron microscopy; ROS, reactive oxygen species; MMP, mitochondrial membrane potential; JC-1, 5,5’,6,6’-tetrachloro-1,1’,3,3’-tetraethylbenzimidazolylcarbocyanine; CCCP, carbonyl cyanide m-chlorophenylhydrazone.

To further elucidate the underlying protective mechanism of Mel against NaF-induced brain injury in offspring rats, this study examined key indicators associated with hippocampal neuron morphology and mitochondrial function. Nissl staining results showed that the number of Nissl bodies in the hippocampal CA1 region was significantly reduced in the NaF group, with some neurons exhibiting pathological changes such as somatic atrophy and nuclear pyknosis; compared with the NaF-exposed group, Mel treatment significantly increased the number of Nissl bodies in the hippocampal CA1 region, indicating that Mel attenuated NaF-induced hippocampal neuronal damage (Figure 4A). The results of transmission electron microscopy revealed that hippocampal neuronal mitochondria in the NaF group exhibited cristae disruption and matrix vacuolization; following Mel treatment, the mitochondrial cristae tended to be intact, and the degree of vacuolization was markedly alleviated (Figure 4B). Mitochondrial function assays indicated that compared with the control group, NaF exposure led to an extremely significant elevation in intracellular ROS levels (Figure 4C,D, *p* < 0.001) and a marked reduction in MMP (Figure 4E,F, *p* < 0.01). Notably, compared with the NaF group, Mel treatment significantly decreased intracellular ROS levels (Figure 4C,D, *p* < 0.05) and markedly restored MMP (Figure 4E,F, *p* < 0.05). These results indicate that Mel effectively attenuates NaF-induced structural and functional damage to hippocampal neuronal mitochondria.

### 3.5. Mel Restores Neuronal Oxidative Phosphorylation and ATP Levels via the SIRT3/HIF-1α Signaling Pathway

**Figure 5:**
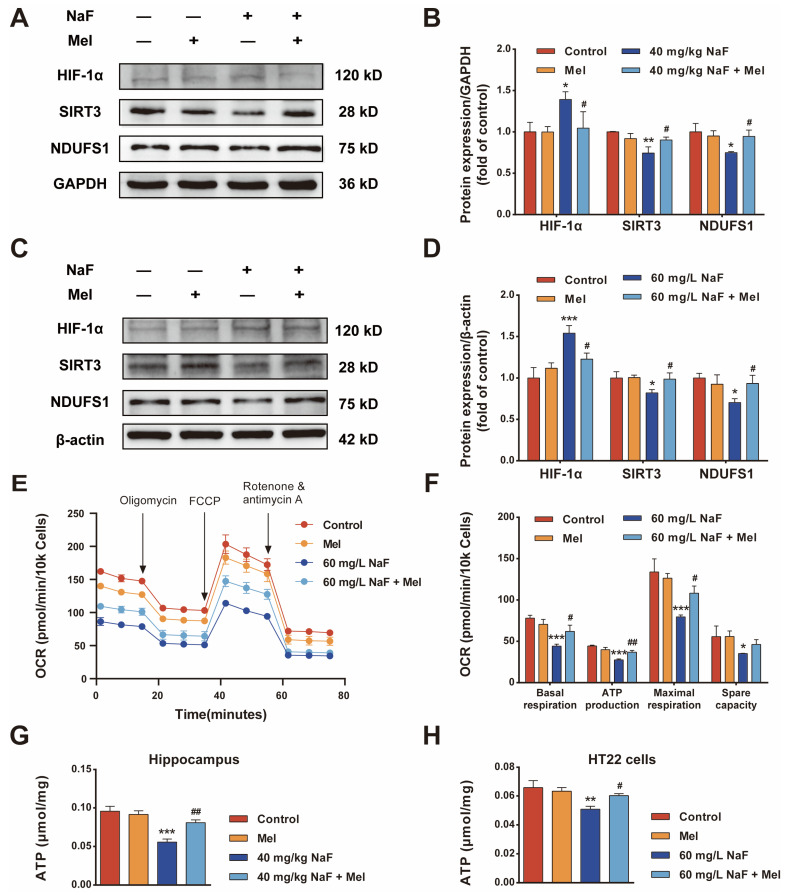
Melatonin restores neuronal oxidative phosphorylation and ATP levels: (**A**) Representative Western blot images of HIF-1α, SIRT3, and NDUFS1 in vivo. (**B**) Quantitative analysis of (**A**). (**C**) Representative Western blot images of HIF-1α, SIRT3, and NDUFS1 in vitro. (**D**) Quantitative analysis of (**C**). (**E**) The OCR of HT22 cells was determined by Seahorse XFe96. (**F**) Quantitative analysis of basal respiration, ATP production, maximal respiration, and spare respiratory capacity in OCR. (**G**) Plot of ATP levels in the hippocampus. (**H**) Plot of ATP levels in HT22 cells. Values represent the mean ± SD (*n* = 3 replicates); * indicates *p* < 0.05, ** *p* < 0.01, *** *p* < 0.001 compared with the control group; ^#^ indicates *p* < 0.05, ^##^ *p* < 0.01 compared with the 40 mg/kg NaF or 60 mg/L NaF group. Mel, melatonin; NaF, sodium fluoride; ATP, adenosine triphosphate; OCR, oxygen consumption rate.

Mitochondria are the primary powerhouses of cells and the key sites for oxidative phosphorylation. Mitochondrial homeostasis is not only essential for cellular energy supply but also critical for sustaining neuronal viability. As a key mitochondrial deacetylase of the Sirtuins family, SIRT3 preserves mitochondrial homeostasis by regulating mitochondrial energy metabolism and intracellular ROS levels [11]. The results demonstrated that compared with the control group, NaF exposure impaired hippocampal neuronal mitochondrial homeostasis (Figure 4) and markedly decreased hippocampal neuronal ATP levels (Figure 5G,H, *p* < 0.05). KEGG enrichment analysis revealed that the HIF-1 signaling pathway is a core pathway mediating the protective effect of Mel against NaF-induced brain injury. As a core energy metabolism regulator, HIF-1α modulates cellular oxidative phosphorylation via glycolytic enzyme activity and mitochondrial function regulation [23]; additionally, elevated intracellular ROS levels markedly upregulate HIF-1α expression [24]. As a core subunit of the mitochondrial respiratory chain complex I, NDUFS1 mediates the critical initial step in oxidative phosphorylation—the central process of cellular energy metabolism. Cellular OCR detected by the Seahorse XFe96 analyzer is an indicator of mitochondrial oxidative phosphorylation status. Western blot results showed that compared with the control group, NaF exposure decreased the protein expression of SIRT3 and NDUFS1 while increasing the protein expression of HIF-1α (Figure 5A–D, *p* < 0.05). Compared with the NaF group, Mel treatment significantly decreased HIF-1α expression and increased SIRT3 and NDUFS1 expression (Figure 5A–D, *p* < 0.05). Furthermore, compared with the control group, NaF exposure markedly reduced the level of cellular oxidative phosphorylation (Figure 5E,F, *p* < 0.05); by contrast, compared with the NaF group, Mel treatment significantly elevated mitochondrial respiratory parameters (basal respiration, ATP production, maximal respiration, and spare respiratory capacity), thereby restoring cellular oxidative phosphorylation (Figure 5E,F, *p* < 0.05) and neuronal ATP levels (Figure 5G,H, *p* < 0.05).

## 4. Discussion

Fluoride, as a ubiquitous environmental pollutant, has attracted global public health attention due to its developmental neurotoxicity induced by excessive exposure. Early-life fluoride exposure can lead to lifelong cognitive deficits, with mechanisms primarily involving oxidative stress, mitochondrial damage, and energy metabolism disorders, yet effective protective strategies are currently lacking [7,8]. This study systematically explored the protective effects and molecular mechanisms of Mel on NaF-induced developmental brain injury through a combination of network pharmacology and experimental validation, confirming for the first time that Mel improves mitochondrial function and energy metabolism by regulating the SIRT3/HIF-1α signaling pathway, thereby alleviating NaF-induced cognitive impairments and providing new theoretical bases and potential targets for the prevention and treatment of fluoride-induced brain damage.

Cognitive impairments represent the core phenotype of NaF-induced developmental neurotoxicity, and the hippocampus—a critical brain region mediating learning and memory—acts as the primary target of fluoride-induced neurotoxic damage [25,26]. The MWM test results of the present study showed that the escape latency of offspring rats exposed to NaF was significantly prolonged, while the time spent in the target quadrant and the number of platform crossings were significantly reduced, indicating that NaF exposure impairs spatial learning and memory abilities in offspring rats, consistent with previous research conclusions [27,28]. Mel intervention significantly reversed cognitive behavioral abnormalities, indicating that Mel exerts a protective effect against NaF-induced cognitive damage. The structural integrity of neurons is fundamentally essential for maintaining brain function at the structural level, and Nissl bodies, as characteristic structures within the neuronal cytoplasm, can objectively reflect the extent of neuronal damage. Long-term ingestion of fluoride-contaminated drinking water (fluoride concentration exceeding 100 mg/L) damages the hippocampus and induces abnormal neuronal morphology [29]; fluoride exposure during pregnancy exacerbates hippocampal damage in offspring in a dose-dependent manner, specifically manifested as a reduction in the number of Nissl bodies and a blurred staining in neurons [30]. This study also found that NaF exposure led to a reduction in the number of Nissl bodies in the CA1 region of the hippocampus, along with neuronal cell body shrinkage and nuclear pyknosis, whereas Mel intervention could increase the number of Nissl bodies and improve neuronal pathological morphology. These findings demonstrate from a morphological standpoint that Mel can mitigate NaF-induced hippocampal neuronal injury, thereby providing structural underpinnings for its neuroprotective and cognitive-protective effects.

Mitochondrial dysfunction is a key mechanism of NaF-induced neurotoxicity, characterized by core features including disruption of mitochondrial ultrastructure, ROS accumulation, MMP decline, and energy metabolism disorders [31]. These pathological changes are interrelated, forming a vicious cycle that exacerbates neuronal damage. Wang D et al. [32] and Xin J et al. [33] reported that NaF exposure impairs the structure and function of hippocampal neuronal mitochondria, which is characterized by excessive ROS accumulation and diminished MMP. This study found that NaF exposure induced mitochondrial cristae fragmentation and matrix vacuolization in hippocampal neurons, disrupting the integrity of mitochondrial structures, whereas Mel intervention significantly restored mitochondrial cristae structure and reduced the degree of vacuolization. The accumulation of ROS can further exacerbate mitochondrial dysfunction, forming a vicious cycle of ROS accumulation and mitochondrial damage [12]. Mel intervention can effectively eliminate ROS and restore mitochondrial membrane potential homeostasis, breaking the vicious cycle and thereby inhibiting further deterioration of mitochondrial function. Impaired energy metabolism is a direct result of mitochondrial dysfunction, and functional abnormality of oxidative phosphorylation—the core of mitochondrial ATP production—causes insufficient neuronal energy supply, leading to neuronal death and cognitive dysfunction [34]. Results from cellular energy metabolism assays demonstrated that NaF exposure significantly reduced cellular oxidative phosphorylation levels, accompanied by decreased ATP content in neurons, while Mel administration significantly enhanced oxidative phosphorylation activity by improving the activity of mitochondrial respiratory chain complex I, thereby restoring neuronal ATP levels and ameliorating neuronal energy deficiency. In neurodegenerative diseases, Mel can protect neurons by inhibiting oxidative stress and inflammatory responses [16]; in models of brain injury induced by environmental toxins, Mel alleviates neuronal damage by maintaining the integrity of mitochondrial structures and restoring mitochondrial function [19]. These previous research findings are consistent with the core findings of the present study that Mel mitigates NaF-induced developmental neurotoxicity by preserving mitochondrial structural integrity, suppressing oxidative stress, and restoring neuronal energy metabolic homeostasis.

To further clarify the molecular mechanisms by which Mel exerts neuroprotective effects, potential targets and signaling pathways were identified via network pharmacology analysis, revealing that the intersection targets of Mel and NaF-induced brain damage are enriched in the HIF-1 signaling pathway, PI3K-AKT signaling pathway, and oxidative stress-related pathways, suggesting that the HIF-1 signaling pathway may be a key target of Mel intervention. HIF-1α, as the core regulatory subunit of the HIF-1 signaling pathway, serves as a critical regulatory hub linking energy metabolism and oxidative stress; concurrently, excessive ROS accumulation can significantly upregulate the expression of HIF-1α, forming a ROS—HIF-1α regulatory axis that exacerbates energy metabolism disorders [23,24]. NaF exposure decreases the expression of p-mTOR and increases intracellular ROS levels in neurons [29]. This study found that Mel-mediated ROS clearance reverses the abnormal activation of the HIF-1 signaling pathway. Existing literature indicates that Mel can suppress HIF-1α protein synthesis by targeting the mTOR signaling pathway [35,36]. As an NAD^+^-dependent mitochondrial deacetylase, SIRT3 preserves mitochondrial homeostasis by deacetylating key target proteins involved in antioxidant defense and energy metabolism [11]. Previous studies have confirmed that Mel upregulates the expression of SIRT3, which in turn deacetylates and activates two core mitochondrial antioxidant enzymes—superoxide dismutase 2 (SOD2) and catalase (CAT) [37]. The activated antioxidant system effectively scavenges excessive accumulated ROS, thereby disrupting the positive feedback loop between ROS overproduction and HIF-1α upregulation. Owing to its amphipathic molecular structure, Mel can directly diffuse into the mitochondrial matrix in a receptor-independent fashion [38]. This characteristic provides a structural basis for its regulation of mitochondrial-related pathways, and this unique mode of regulation also offers a novel perspective for elucidating the upregulatory effect of Mel on SIRT3 expression in developmental neurotoxicity models. The present study found that NaF exposure significantly downregulated SIRT3 protein expression and upregulated HIF-1α protein expression, while Mel intervention could significantly reverse the abnormal expression of these proteins and restore mitochondrial oxidative phosphorylation, suggesting that Mel may exert its effects by regulating the SIRT3/HIF-1α signaling pathway. Previous studies have demonstrated that the activation of SIRT3 can mitigate fluoride-induced mitochondrial damage [11], while Mel may activate mitochondrial antioxidant enzyme activity and enhance mitochondrial energy metabolism through activating [39]. This further confirms the central regulatory role of the SIRT3/HIF-1α signaling pathway in fluoride-induced neurotoxicity. This comprehensive regulatory network not only explains the restoration of mitochondrial oxidative phosphorylation capacity and neuronal ATP supply that was observed in this experiment but also provides a mechanistic basis for the long-term neuroprotective effects of Mel in developmental brain injury. Notably, mechanisms such as direct mitochondrial targeting and epigenetic regulation by Mel highlight its unique advantages as a neuroprotective agent—its ability to target multiple subcellular compartments and exert sustained regulatory effects, which cannot be achieved by conventional antioxidants.

## 5. Conclusions

In summary, this study combined network pharmacology and experimental validation to confirm that Mel exerts significant neuroprotective effects against NaF-induced developmental brain injury, mainly by improving mitochondrial dysfunction and energy metabolism disorders. The core mechanism lies in the fact that Mel regulates the SIRT3/HIF-1α signaling pathway, clears excessive ROS, and stabilizes MMP, thereby restoring mitochondrial homeostasis and oxidative phosphorylation levels, ultimately alleviating neuronal damage and cognitive impairment. These findings provide novel insights into the mechanisms of fluoride-induced neurotoxicity and indicate that Mel holds promise as a potential candidate drug, thereby laying a solid foundation for the development of novel protective regimens against fluoride-induced brain damage.

## Data Availability

The original contributions presented in this study are included in the article and Supplementary Material. Further inquiries can be directed to the corresponding author.

## Supplementary Materials

The following supporting information can be downloaded at https://www.mdpi.com/article/10.3390/toxics14020128/s1, Figure S1: The CCK-8 experiment screened melatonin doses: (A) The effect of melatonin on the viability of HT22 cells. (B) Effect of combined melatonin and NaF treatment on the viability of HT22 cells. Values represent the mean ± SD (*n* = 3 replicates). *** *p* < 0.001 compared with the control group; # *p* < 0.05 compared with the 60 mg/L NaF group; Table S1: gene symbol; Table S2: gene symbol.

## References

[1] Bittencourt L.O., Dionizio A., Ferreira M.K.M., Aragão W.A.B., de Carvalho Cartágenes S., Puty B., do Socorro Ferraz Maia C., Zohoori F.V., Buzalaf M.A.R., Lima R.R. (2023). Prolonged exposure to high fluoride levels during adolescence to adulthood elicits molecular, morphological, and functional impairments in the hippocampus. Sci. Rep..

[2] Kumar P., Kumar M., Barnawi A.B., Maurya P., Singh S., Shah D., Yadav V.K., Kumar A., Kumar R., Yadav K.K. (2024). A review on fluoride contamination in groundwater and human health implications and its remediation: A sustainable approaches. Environ. Toxicol. Pharmacol..

[3] Ma Y., Meng X., Sowanou A., Wang J., Li H., Li A., Zhong N., Yao Y., Pei J. (2023). Effect of Fluoride on the Expression of 8-Hydroxy-2′-Deoxyguanosine in the Blood, Kidney, Liver, and Brain of Rats. Biol. Trace Elem. Res..

[4] Wei Y.L., Lin X.C., Liu Y.Y., Lei Y.Q., Zhuang X.D., Zhang H.T., Wang X.R. (2024). Effects of water fluoridation on early embryonic development of zebrafish. Ecotoxicol. Environ. Saf..

[5] Zeng Y., Stevens G.W.J.M., Helbich M. (2023). Longitudinal associations of neighbourhood environmental exposures with mental health problems during adolescence: Findings from the TRAILS study. Environ. Int..

[6] Goodrich J.A., Alderete T.L., Baumert B.O., Berhane K., Chen Z., Gilliland F.D., Goran M.I., Hu X., Jones D.P., Margetaki K. (2021). Exposure to Perfluoroalkyl Substances and Glucose Homeostasis in Youth. Environ. Health Perspect..

[7] Taylor K.W., Eftim S.E., Sibrizzi C.A., Blain R.B., Magnuson K., Hartman P.A., Rooney A.A., Bucher J.R. (2025). Fluoride Exposure and Children’s IQ Scores: A Systematic Review and Meta-Analysis. JAMA Pediatr..

[8] Zhang Y., Gao Y., Liu X. (2024). Focus on cognitive impairment induced by excessive fluoride: An update review. Neuroscience.

[9] Chen L., Jia P., Liu Y., Wang R., Yin Z., Hu D., Ning H., Ge Y. (2023). Fluoride exposure disrupts the cytoskeletal arrangement and ATP synthesis of HT-22 cell by activating the RhoA/ROCK signaling pathway. Ecotoxicol. Environ. Saf..

[10] Wei M., Ye Y., Ali M.M., Chamba Y., Tang J., Shang P. (2022). Effect of Fluoride on Cytotoxicity Involved in Mitochondrial Dysfunction: A Review of Mechanism. Front. Vet. Sci..

[11] Wang D., Cao L., Zhou X., Wang G., Ma Y., Hao X., Fan H. (2022). Mitigation of honokiol on fluoride-induced mitochondrial oxidative stress, mitochondrial dysfunction, and cognitive deficits through activating AMPK/PGC-1α/Sirt3. J. Hazard. Mater..

[12] Boras M.M., Krulj V., Karahmet A., Omerbasic K., Nawaz A., Pavković D., Sher E.K. (2025). Oxidative Stress-Induced mechanisms in neurodegeneration and Eryptosis: Implications for neurological and systemic disorders. Brain Res..

[13] Javanbakht P., Talebinasab A., Asadi-Golshan R., Shabani M., Kashani I.R., Mojaverrostami S. (2025). Effects of Quercetin against fluoride-induced neurotoxicity in the medial prefrontal cortex of rats: A stereological, histochemical and behavioral study. Food Chem. Toxicol..

[14] Trejo-Solís C., Rojas-Tomé I.S., Jung-Cook H., Palomares-Alonso F. (2025). Melatonin combined with antineoplastic drugs or natural products for cancer treatment: An update. Curr. Res. Pharmacol. Drug Discov..

[15] Bocheva G., Bakalov D., Iliev P., Tafradjiiska-Hadjiolova R. (2024). The Vital Role of Melatonin and Its Metabolites in the Neuroprotection and Retardation of Brain Aging. Int. J. Mol. Sci..

[16] Deepika, Thakur A., Panghal A., Pundir R., Singh C., Goyal M., Namdeo A.G., Kumar J. (2025). Crosstalk between copper, Alzheimer’s disease, and Mel. Biometals.

[17] Guo Y., Liu C. (2025). Mel attenuates MPP(+)-induced autophagy via heat shock protein in the Parkinson’s disease mouse model. PeerJ.

[18] El Brouzi M.Y., Lamtai M., Zghari O., El Hamzaoui A., Rezqaoui A., Hadch Z., Fath N., Ouichou A., El Hessni A., Mesfioui A. (2024). Mel is a Neuroprotective and Antioxidant Agent against Neurotoxicity Induced by an Intrahippocampal Injection of Nickel in Rats. Neurotox. Res..

[19] Dong L., Sun Q., Qiu H., Yang K., Xiao B., Xia T., Wang A., Gao H., Zhang S. (2023). Mel protects against developmental PBDE-47 neurotoxicity by targeting the AMPK/mitophagy axis. J. Pineal Res..

[20] Xu P., Xing H., Ma Y., Ding X., Li T., Zhang Y., Liu L., Ma J., Niu Q. (2025). Fluoride Induces Neurocytotoxicity by Disrupting Lysosomal Iron Metabolism and Membrane Permeability. Biol. Trace Elem. Res..

[21] Zeng L., He J., Liu C., Zhang F., Zhang Z., Chen H., Wang Q., Ding X., Luo H. (2022). Mel Attenuates Ropivacaine-Induced Apoptosis by Inhibiting Excessive Mitophagy Through the Parkin/PINK1 Pathway in PC12 and HT22 Cells. Inflammation.

[22] Zhou Y., Zhou B., Pache L., Chang M., Khodabakhshi A.H., Tanaseichuk O., Benner C., Chanda S.K. (2019). Metascape provides a biologist-oriented resource for the analysis of systems-level datasets. Nat. Commun..

[23] Bao X., Zhang J., Huang G., Yan J., Xu C., Dou Z., Sun C., Zhang H. (2021). The crosstalk between HIFs and mitochondrial dysfunctions in cancer development. Cell Death Dis..

[24] Xueqiang Z., Yu Z., Wendong L., Shuying F., Jie H. (2025). Salidroside Alleviates Renal Ischemia-reperfusion Injury by Inhibiting Macrophage Pyroptosis Through HIF Signaling. Inflammation.

[25] Veneri F., Vinceti M., Generali L., Giannone M.E., Mazzoleni E., Birnbaum L.S., Consolo U., Filippini T. (2023). Fluoride exposure and cognitive neurodevelopment: Systematic review and dose-response meta-analysis. Environ. Res..

[26] Denoth-Lippuner A., Jessberger S. (2021). Formation and integration of new neurons in the adult hippocampus. Nat. Rev. Neurosci..

[27] Zhang J., Xu P., Zhang Y., Li T., Ding X., Liu L., Yao P., Niu Q. (2025). Unraveling the role of abnormal AMPK and CRMP-2 phosphorylation in developmental fluoride neurotoxicity: Implications for synaptic damage and neurological disorders. Ecotoxicol. Environ. Saf..

[28] Du Y., Feng Z., Gao M., Wang A., Yan X., Chen R., Liu B., Yu F., Ba Y., Zhou G. (2024). Impaired neurogenesis induced by fluoride via the Notch1 signaling and effects of carvacrol intervention. Environ. Pollut..

[29] Ran L.Y., Xiang J., Zeng X.X., He W.W., Dong Y.T., Yu W.F., Qi X.L., Xiao Y., Cao K., Zou J. (2023). The influence of NQO2 on the dysfunctional autophagy and oxidative stress induced in the hippocampus of rats and in SH-SY5Y cells by fluoride. CNS Neurosci. Ther..

[30] Xu W., Hu Z., Tang Y., Zhang J., Xu S., Niu Q. (2023). Excessive Lysosomal Stress Response and Consequently Impaired Autophagy Contribute to Fluoride-Induced Developmental Neurotoxicity. Biol. Trace Elem. Res..

[31] Mishra J., Bhatti G.K., Sehrawat A., Singh C., Singh A., Reddy A.P., Reddy P.H., Bhatti J.S. (2022). Modulating autophagy and mitophagy as a promising therapeutic approach in neurodegenerative disorders. Life Sci..

[32] Wang D., Cao L., Pan S., Wang G., Wang L., Cao N., Hao X. (2021). Sirt3-mediated mitochondrial dysfunction is involved in fluoride-induced cognitive deficits. Food Chem. Toxicol..

[33] Xin J., Zhu B., Wang H., Zhang Y., Sun N., Cao X., Zheng L., Zhou Y., Fang J., Jing B. (2023). Prolonged fluoride exposure induces spatial-memory deficit and hippocampal dysfunction by inhibiting small heat shock protein 22 in mice. J. Hazard. Mater..

[34] Liu J.C., Zhao X.Y., Wu M.L., Shi Y.F., Huang Z.P., Fang L.P., Zhu C., Peng X., Shi Z.L., Lan L.J. (2024). GPR50 regulates neuronal development as a mitophagy receptor. Cell Death Dis..

[35] Xue K.H., Jiang Y.F., Bai J.Y., Zhang D.Z., Chen Y.H., Ma J.B., Zhu Z.J., Wang X., Guo P. (2023). Melatonin suppresses Akt/mTOR/S6K activity, induces cell apoptosis, and synergistically inhibits cell growth with sunitinib in renal carcinoma cells via reversing Warburg effect. Redox Rep..

[36] Prieto-Domínguez N., Méndez-Blanco C., Carbajo-Pescador S., Fondevila F., García-Palomo A., González-Gallego J., Mauriz J.L. (2017). Melatonin enhances sorafenib actions in human hepatocarcinoma cells by inhibiting mTORC1/p70S6K/HIF-1α and hypoxia-mediated mitophagy. Oncotarget.

[37] Wang L., Wu L., Wang T., Yue Y., Jiang Z., Jiang P., Zhou H., He L., Xia Z., Song Y. (2026). Melatonin ameliorates copper accumulation-induced cognitive impairment in Wilson disease via activation of the SIRT3/FOXO3α signaling pathway. Neuropharmacology.

[38] Volpe O.A.J., Zuccari D., Chuffa L.G.A., Reiter R.J. (2025). Melatonin and Lipid Peroxidation: Antioxidant Shield and Therapeutic Potential. Front. Biosci. (Landmark Ed).

[39] Ge X., Wang C., Yang G., Maimaiti D., Hou M., Liu H., Yang H., Chen X., Xu Y., He F. (2024). Enhancement of mitochondrial energy metabolism by Mel promotes vascularized skeletal muscle regeneration in a volumetric muscle loss model. Free Radic. Biol. Med..

