# Supplementary material for: Combining Network Pharmacology and Experimental Verification to Investigate the Protective Effect of Melatonin on Fluoride-Induced Brain Injury
Source: Toxics. 2026 Jan 29;14(2):128. doi: 10.3390/toxics14020128 (PMC12944959; doi:10.3390/toxics14020128)
Supplement: Supplementary file 1 [file toxics-14-00128-s001.zip › supplement Figure S1.pdf]

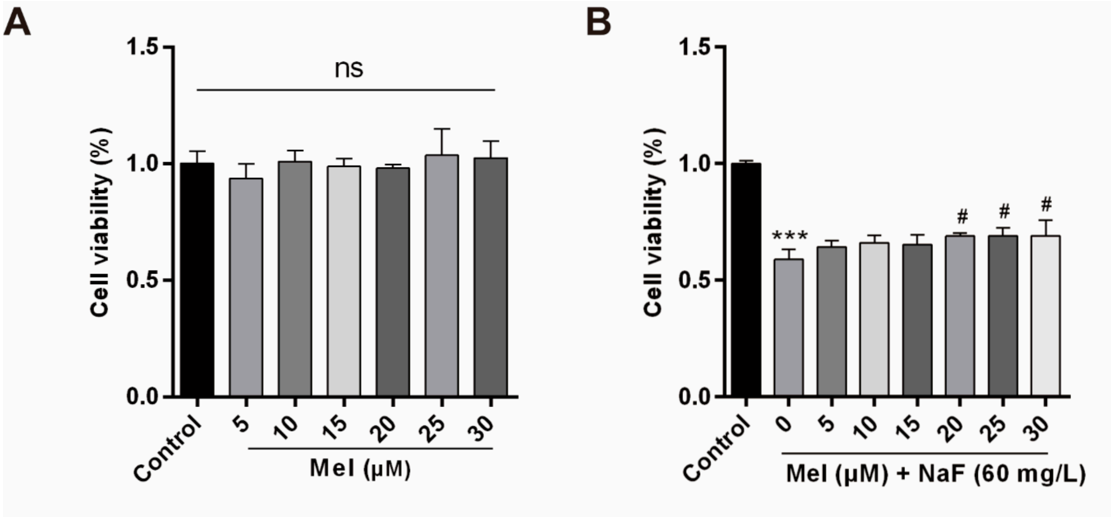

Figure S1. CCK-8 experiment screened melatonin doses. (A) The effect of melatonin on the viability of HT22 cells. (B) Effect of combined melatonin and NaF treatment on the viability of HT22 cells. Values represent the mean ± SD (n = 3 replicates). $^{***}P <$ 0.001 compared with the control group; $^{\#}P < 0.05$ compared with the 60 mg/L NaF group.
